# Supplementary material for: The relationship between musical training and the processing of audiovisual correspondences: Evidence from a reaction time task
Source: PLoS One. 2023 Apr 6;18(4):e0282691. doi: 10.1371/journal.pone.0282691 (PMC10079049; doi:10.1371/journal.pone.0282691)
Supplement: Attachment — Submitted filename: Response to Reviewers.docx [file pone.0282691.s001.docx]

**Response to Reviewers**

**Reviewer #1 comments to the authors**

This paper investigates the effect of musicianship on three audiovisual crossmodal correspondences: pitch-elevation/symbolic & non-symbolic magnitude (PE, PSM, PNSM, respectively). Musicianship had no effect on reaction times; but musicians were more accurate than non-musicians overall, largely because they were more accurate on incongruent trials. Accuracy was highest on the PE task but there appear to be no differences between musicians and non-musicians on any task. Similarly, there is no group effect or group/task interaction for speed-accuracy scores. The authors attribute these results to top-down effects on pre-attentive processing.

While this is an interesting question, it’s quite hard to work out what participants were actually asked to do and, thus, how the question is being addressed.

**Response to reviewer:** *The authors would like to sincerely thank the reviewer for the feedback, and especially for highlighting the weaknesses and raising the questions on issues that were not clearly communicated or considered in the original manuscript. We find the feedback very constructive, and have taken the utmost care to address your concerns in the revised manuscript.*

*As for making it more clear what the participants were asked to do, we have added an additional figure in the manuscript (figure 2) that shows the experimental procedure in detail in congruent and incongruent conditions for each of the three audiovisual stimulus categories. With the figure we have added the following legend: “****Fig 2.*** *Examples of the experimental procedure in both congruent and incongruent conditions for each of the three audiovisual stimulus category. Panels* ***A – B*** *illustrate congruent and incongruent trials in the pitch-elevation category, respectively. Each panel/trial consisted of a single video with the total length of 860 ms. In this video clip the participant saw two visual stimuli each associated with a sound on a particular pitch. The pitch varied in frequency and the visual stimulus varied in elevation. Congruency was estimated according to an explicitly learned rule, “The higher the spatial elevation, the higher the tone”. Panels* ***C – D*** *illustrate congruent and incongruent trials in the symbolic magnitude-pitch category, respectively. Here, the visual stimulus varied in value of the number shown and congruency was estimated according to the explicitly learned rule, “the higher the number presented, the higher the tone”. Panels* ***E – F*** *illustrate congruent and incongruent trials in the nonsymbolic magnitude-pitch category, respectively. Here, the visual stimulus varied in the number of circles shown and congruency was estimated according to the explicitly learned rule, “the more dots presented, the higher the tone”. Note that in each category congruency/incongruency could be induced either with the visual stimuli or with the auditory stimuli.”*

1. Each trial consists of two audiovisual pairs, let’s say 1 dot and 4 dots each accompanied by a different tone. What exactly makes a trial congruent or incongruent? If the tones are different in each pair, one has to be higher than the other. Is a congruent trial one in which the single dot is accompanied by a low tone and the 4 dots by a high tone, with an incongruent trial being 1-dot/high tone plus 4-dots/low tone? If so, what’s the point of the pairs? You could achieve (in)congruency with a single audiovisual stimulus, i.e. congruent = 1-dot/low tone, incongruent = 1-dot/high tone. Or do participants have to say whether the second pair breaks the rule?

**Response to reviewer:** *We do acknowledge that the task the participants were asked to do could have been communicated more clearly. Here, congruency/incongruency was defined by reflecting whether the abstract rule was followed or not. The reviewer is correct in that a congruent trial is one in which e.g. a single dot is accompanied by a low tone followed by 4 dots accompanied by a high tone. An incongruent trial, on the other hand, could be one in which a single dot accompanied by a high tone is followed by 4 dots accompanied by a low tone. One of such sequence formed a single trial and consisted of a pair of audiovisual stimuli (one dot with a tone and 4 dots with a tone). After such a pair was shown to the participant, the participants were instructed to indicate whether the rules were followed or not.*

*It is not clear how such a reflection of the congruency (e.g. whether the rules were followed or not) could be achieved with a single audiovisual stimulus (i.e. one dot with a low tone). We do feel that the confusion here is due to our poor communication of the task (and what is meant by ‘a pair of audiovisual stimuli’). Hence, we have made the following changes to the corresponding parts of the manuscript:*

*We have added additional figure (figure 2) showing the detailed experimental procedure in congruent and incongruent conditions for each of the tree audiovisual stimulus categories. With the figure, we have written a detailed legend explaining the figure (and hence the procedure) in what we believe to be clear terms (see also reply above). Moreover, we have edited the methods section to explain the procedure more clearly (lines 250-290).*

*Lines 250-290 now say the following: “Next, the experiment began. The experimental procedure is shown in Fig 2.*

*Enter figure 2 here*

***Fig 2. Examples of the experimental procedure in both congruent and incongruent conditions for each of the tree audiovisual stimulus category.*** *Panels* ***A – B*** *illustrate congruent and incongruent trials in the pitch-elevation category, respectively. Each panel/trial consisted of a single video with the total length of 860 ms. In this video clip, the participant saw two visual stimuli each associated with a sound on a particular pitch. The pitch varied in frequency and the visual stimulus varied in elevation. Congruency was estimated according to an explicitly learned rule, “The higher the spatial elevation, the higher the tone”. Panels* ***C – D*** *illustrate congruent and incongruent trials in the symbolic magnitude-pitch category, respectively. Here, the stimulus varied in value of the number shown and congruency was estimated according to the explicitly learned rule, “the higher the number presented, the higher the tone”. Panels* ***E – F*** *illustrate congruent and incongruent trials in the non-symbolic magnitude-pitch category, respectively. Here, the stimulus varied in the number of circles shown and congruency was estimated according to the explicitly learned rule, “the more dots presented, the higher the tone”. Note that in each category congruency/incongruency could be induced either with the visual or with the auditory part of the stimuli.*

*Each trial consisted of a single video clip (a single panel on Fig 2). The sequence of events in each trial was as follows: the first audiovisual stimulus was presented in the middle of the screen for 400 ms, followed by a short break of 60 ms before the second audiovisual stimulus (400 ms). Each of the audiovisual stimuli consisted of a visual picture (see Fig 1) associated with an auditory stimulus that varied in pitch. The onset time for the auditory stimuli was synchronized with the onset of the visual images. After presenting both audiovisual stimuli (i.e. after presenting a single trial consisting of a pair of audiovisual stimuli), congruency was estimated; the pair of audiovisual stimuli allowed the participants to estimate congruency between the stimuli based on the explicitly learned abstract rules. These rules were “The higher the spatial elevation, the higher the tone”, “the more dots presented, the higher the tone”, and “the higher the number presented, the higher the tone”, depending on the category. The participant were instructed to respond as quickly and accurate as possible with their right hand only by pressing ‘K’ on the keyboard when the corresponding rules were followed, and pressing the button ‘L’ when the rules were not followed. After the response, a 1000 ms blank screen appeared before the next trial. The researcher further instructed the participants verbally, and made sure the instructions were understood properly.*

*There were two experimental blocks, and each block consisted of 180 trials. In each block, there were three audiovisual stimulus categories with 60 trials in each (30 congruent and 30 incongruent). The order of the trials was pseudo-randomized across the participants so that there were not consecutive trials having the same stimulus type. This randomization process also enabled the elimination of any potential bias caused by varying intervals between the auditory tones and the visual stimuli.”*

2. It would be helpful to amend Fig 1 to indicate how the tones are paired with the visuals (e.g., insert text ‘high’/‘low’), and to show both congruent and incongruent examples.

**Response to reviewer:** *Please, see the above response.*

3. PE, PSM and PNSM trials are randomly ordered within a block of 180 trials, so participants have to remember three different rules and switch between them at random. To what extent do the results simply reflect task demands and switching costs?

**Response to reviewer:** *We would like to thank the reviewer for this interesting suggestion. Indeed the task employed involved a “judgment” switching component, but please notice that the task did not require shifting of the response mapping, which remained the same across the judgment conditions. According to Von Bastian, & Druey (2017), who systematically investigated all types of switching (judgment, dimension, stimulus, mapping and response), the response mapping shifting is the one which is central and most relevant to switching ability and switching cost. In addition, and given that RTs is the most sensitive measure of switching cost, and that the main effect of group was not significant (and this factor did not interact with the other factors) in the response time analyses it is unlikely that a better switching ability in musicians could explain our results.*

*We discuss the cognitive demand of the task and its possible effects on the results in lines 440-496. This section also includes a paragraph specifically discussion the possible effect of task switching and our reponse to it.*

4. Incidentally, the paper constantly refers to ‘visual stimuli’ and ‘visual stimulus categories’ when they are, in fact, audiovisual. In any case, what the authors seem to mean by this is the three crossmodal correspondences/tasks, it would be clearer to refer to them as such.

**Response to reviewer:** *Thank you for this comment.. We edited the MS to increase clarity. For example, in the beginning of section 2.2. Stimuli, instead of talking about “three types of visual stimuli” we know refer to the visual and auditory parts of the audiovisual stimuli. Similarly, in the legend of figure 1, we refer to just stimulus, instead of “visual stimulus”. Similar changes have been made in the manuscript where necessary.*

5. There also seems to be some confusion over what constitutes congruency: better performance in a multisensory condition than a unisensory condition reflects integration, not congruency (p5); see also comments on the Discussion.

**Response to reviewer:** *We do acknowledge the confusion in the use of the terms here. This part has been edited to say (lines 105-109): “There is a relatively large number of reaction time studies done with non-musicians in the context of multimodal sensory integration. These investigations have consistently reported better performance with multimodal stimuli: faster detection of the target in the multimodal condition relative to unimodal condition (see for example Miller, 1991; for reviews see Parise & Spence, 2013; Spence, 2011).*

**RESULTS**

6. 3.1 Please report the t-tests for the post-hoc comparisons for the main effect of task (and the Bonferroni-corrected alpha). Was the group/task interaction not significant? It would be helpful to add an overall mean to the bottom of Table 1 so that the reader can connect back to the text.

**Response to reviewer:** *We have added more information on paragraph 3.1. We now explicitly state that the interactions were statistically non-significant and that the post hoc comparison was made using the estimated marginal means, and not with t-tests. The reported p-values for the estimated marginal means are bonferroni corrected.*

*We have also added the overall means in table 1, as per the suggestion.*

7. 3.2 What does Figure 2 show? Does it refer to performance against chance (p14) – in which it should show what value would represent chance and also show median values as in the text instead of changing to mean values – or does it reflect the Mann-Whitney test (p15)?

The legend suggests that it refers to “musicians and non-musicians discriminating between congruent and incongruent trials”. This would be more useful than either of the options above but, in that case, the data would change to number of mistakes and for each group should be broken down by trial type. This is the “large advantage for musicians” (p18/367) so let’s see that clearly.

**Response to reviewer:** *We acknowledge that indeed it was unclear to what figure 3 (figure 2 in previous version) is referring to. It shows the difference in the d-primes between musicians and non-musicians discriminating between congruent and incongruent stimuli. To make this clearer, we have added the following sentence on paragraph 3.2. Accuracy: Fig 3 shows the difference in the mean d-prime between musicians and non-musicians discriminating between congruent and incongruent stimuli.*

*As for why the d-prime is calculated combining the audiovisual stimulus categories, as we responded to reviewer #2 (corrections number 6.), when recording the mistakes, we had several participants who made zero mistakes in some of the audiovisual categories, especially with congruent stimuli, hence causing issues in the d-prime calculations. As our main interest lied in the potential difference between musicians and non-musicians, rather than between the types of visual stimuli, to overcome the issue caused by participants with zero mistakes, we followed the suggestions of Stanislaw & Todorov (1999) and combined the data from several categories before calculating the hit and false-alarm rates (*[*https://link.springer.com/content/pdf/10.3758/BF03207704.pdf*](https://link.springer.com/content/pdf/10.3758/BF03207704.pdf)*). Consequently, when we investigated the accuracy between the visual categories further, we used the raw number of mistakes in the analyses.*

*We have added this information also in paragraph 2.4. Analysis which now states “As our main interest lied in the potential difference in accuracy between musicians and non-musicians and as several participants made zero mistakes in some of the stimulus categories, instead of calculating the d-prime for each audiovisual category individually, following the suggestions of Stanislaw & Todorov (1999), we combined the data from the three stimulus categories before calculating the hit and false-alarm rates.* *For the further investigation of audiovisual category-wise accuracy, we used the raw number of mistakes.”*

8. The final paragraph of 3.2 describes the main effect of trial type and would be better placed after the paragraph reporting the main effect of group.

Were there any group differences on each task?

**Response to reviewer:** *We have moved the paragraph below the results describing the difference in raw mistakes between musicians and non-musicians, as suggested. However, we kept the results with d-primes and raw mistakes separate.*

*We have also added a paragraph describing the difference between musicians and non-musicians in each of the audiovisual categories individually (lines 341-346). Correspondingly, we added these results also in the discussion section (lines 390-392 & 407-409).*

9. 3.3 Please report the means, SDs, tests, p-values, and corrected alpha for the explanation of the task/trial type interaction.

Was the group/task interaction not significant?

**Response to reviewer:** *For the interactions in reaction times, we have the results from the ANOVA with estimated marginal means, however, we feel reporting the estimated marginal means (with SDs, p-values, and corrected p-values) is not crucial, since the results cannot be separated from chance under the null hypothesis with a high enough precision. Rather, we have explicitly stated that the interactions did not reach statistical significance, and provided the level above which all the p-values lay.*

**DISCUSSION**

10. Paragraph 1 needs to make clear that the advantage for musicians is quite general, across all tasks, with no group/task interactions (I assume, see comments above).

**Response to reviewer:** *See our response above (correction #8).*

11. The opening sentence of paragraph 2 is a bit misleading – the results show *overall* main effects of congruency (faster RTs, fewer mistakes, smaller LISAS for congruent compared to incongruent) but no congruency/task interactions are reported for RTs or accuracy: presumably these were not significant? There is such an interaction in the LISAS analysis but, instead of a difference between congruent and incongruent trials *within* a task (i.e., a congruency effect), this seems to reflect differences in the congruent/incongruent conditions *between* tasks (and only the magnitude tasks) which is not helpful. Just because overall RTs are faster for the PE task compared to the other two doesn’t mean there’s a congruency effect.

**Response to reviewer:** *This is true: reading the sentence did give a misleading impression. What we meant was that when measured with RTs, d-primes, number of mistakes and LISAS, we found a congruency effect. We have corrected this sentence to state “Specifically, our results showed significant main effects of congruency with all measures (response times, d primes, number of mistakes, and linear integrated speed-accuracy scores), suggesting audiovisual integration for all three stimulus categories.”*

*Moreover, we have re-structured and largely re-written the entire discussion section to better reflect the comments and suggestions by both of the reviewers. Hence, the entire discussion section should be re-visited.*

**MINOR POINTS**

12. p6/138: after ‘sets’ insert ‘out’ - *corrected*

p8: the first paragraph should be moved to the start of section 2.4 where it makes more sense; the title for section 2.1 then becomes just ‘Participants’. - *corrected*

p12/260: if there are 2 experimental blocks each with 180 trials then all stimuli are presented twice? *– This was indeed a bit unclear. It now states “There were two experimental blocks, and each block consisted of 180 trials. In each block, there were three audiovisual stimulus categories with 60 trials in each (30 congruent and 30 incongruent).*” *(lines 255-257).*

p15/324, 333, 334: please report the statistical test supporting the p-value. - *corrected*

p16/344-345: the p-values are redundant, they just reflect the main effect reported a few lines above. – *removed*

p16/346-348: please report the tests underlying the p-values and the corrected alpha level. - *corrected*

p12/262: ‘trials’ not ‘trails’ - *corrected*

p18/376, p19/400: ‘innateness’ would be better. - *corrected*

p19/391: I’m not sure I would refer to the RT data as ‘raw’ because they were considerably cleaned up – perhaps ‘absolute’ or just not qualify it at all. - *corrected*

p20/421: after ‘previous’ insert ‘studies’. - *corrected*

Figure 1: the labels for symbolic and non-symbolic magnitude need to be swapped so that they are under the correct task. - *corrected*

**Reviewer #2 comments to the authors**

The current study examined the influences of musical training on crossmodal correspondences between vision and audition. Three rules of correspondences were tested: pitch-elevation, pitch-numerosity, and pitch-digit pairings. The results demonstrated the only effect involves musical training was that musicians responded less errors than non-musicians, especially in the incongruent trials. In addition, responses were faster and less errors in the congruent than in the incongruent condition, and faster for the pitch-elevation pairing than for the pitch-numerosity and pitch-digit pairings; similar effect was demonstrated when considering both response time and errors using the index of LISAS.

In general, the rationale and the design of the study is confusing, so it is hard for me to reach any clear conclusion. Here are my main concerns:

**Response to reviewer:** *The authors would like to sincerely thank the reviewer for the feedback. We find the feedback very constructive, especially the feedback concerning clarity and the rationale for parts of the manuscript. We have taken the utmost care to address your concerns in the revised manuscript.*

1. The first concern is the rationale of using accuracy and response time measures. To my knowledge, accuracy is more suitable than response time when probing early processing of stimuli with time-limited presentation (Norman & Bobrow, 1975, Cognitive Psychology; Santee & Egeth, 1982, JEP:HPP). In contrast to the authors’ arguments, response time measure often involves the accumulation process of decision making.

**Response to the reviewer:** *We wish to thank the reviewer for pointing this out, and we fully agree with the notion that reaction times and accuracy measures tap onto different underlying processes. As such, each type of response yields different information, with reaction time reflecting more on implicit processing and accuracy more on explicit processing. This is indeed what we argue in the manuscript, and provide as a justification for incorporating both of these two measures; we aim to capture these differences by using both measures.*

*We have edited the wording throughout the manuscript to better reflect this comment and removed the parts of text explicitly referring to ‘requirements of conscious processing’ (lines 99-110). Instead, we now highlight the point that the two measures reflect different cognitive processes (implicit/explicit), and hence, provide complementary information of the underlying processes.*

*Moreover, we have re-structured and largely re-written the entire discussion section to better reflect the comments and suggestions by both of the reviewers. The discussion section now includes parts discussing the results in relation to these two measures and the underlying cognitive processing. Hence, we recommend that the entire discussion section should be re-visited.*

2. It is unclear how to separate different types of crossmodal correspondences at pre-attentive stage associated with sub-cortical structure versus higher-order cognitive process. Presumably, sub-cortical structures mention by the authors (superior colliculus) does not represent stimulus identity, and therefore it is not possible to reveal any crossmodal correspondences at this level of processing.

**Response to the reviewer:** *We agree with the reviewer that behaviourally the distinction between higher order cortical processing and lower level sub-cortical processing is difficult, in not directly impossible. However, this was not the aim of the manuscript. Rather, we in the corrected manuscript speculate an interplay between top-down, higher order and pre-attentive processing, and provide some suggestion on how future studies could potentially break down this interaction further.*

*That said, a great line of research has been done on multisensory correspondences (Bidelman, 2016; Laundry & Champoux, 2017; Miller, 1991; Paraskevopoulos et al., 2014; Paraskevopoulos et al., 2012; Parise & Spence, 2013; Spence, 2011) and previous research has already identified the role of sub-cortical structures, including but not limited to the superior colliculi in the processing of such stimuli (e.g. Stein & Meredith, 1993; for a review see Alais, Newell & Mamassian, 2010). The mentioning of these structures in the manuscript is hence grounded on a-priori knowledge provided by such relevant literature.*

*Moreover, we have re-structured and largely re-written the entire discussion section to better reflect the comments and suggestions by both of the reviewers. Hence, the entire discussion section should be re-visited.*

3. It is unclear why the three correspondences rules for participants were defined as “newly learned”—did the participants truly learn the rule, or they were merely instructed to responded in such ways?
More specifically the rule “The higher the spatial elevation, the higher the tone” is a natural correspondence and has been repeatedly reported in literature (reviewed in Discussion). However, the other two rules “the more dots presented, the higher the tone” and “the higher the number presented, the higher the tone” seem to be counter-intuitive to the vertical numerical line (Hung, Hung, Tzeng, & Wu, 2008, Cognition). It is therefore not surprising that the response time for the first rule was faster than the latter two rules.

**Response to the reviewer:** *We fully agree that the advantageous effect of pitch-elevation over pitch-symbolic magnitude and pitch-non-symbolic magnitude in and of itself is not surprising. Indeed, in the manuscript we do not claim that it is, and instead, we relatively shortly discuss it in relation to previous papers suggesting its innateness and more extensive experience in comparison to the two other types of audiovisual stimuli used in the experiment (the two magnitude categories). Indeed, precisely because of pitch-elevation has been suggested to be a naturally occurring statistic with which people in general – and not only musicians – have more extensive experience, we added the two other audiovisual categories. With pich-magnitude and pitch-non-symbolic magnitude – as we discuss in the paper – such a natural linkage is not that clear (although see the manuscript discussion section lines 544 – 558).*

*Hence, our task also requires the participants to estimate congruence based on explicitly learned rule, binding other unrelated unisensory stimuli. Here, ‘newly/explicitly’ is particularly true with the two magnitude categories, as no natural linkage between magnitudes and pitch has been demonstrated, and by ‘learning’ we simply refer to the participants’ internalizing the rule, keeping it in mind, and discriminating the audiovisual stimuli accordingly.*

*In addition to not having a clear, naturally occurring linkage with pitch, magnitude processing in and of itself has been argued to reflect “higher” cognitive processes (Dehaene, Piazza, Pinel & Cohen, 2003; Paraskevopoulos et al., 2014). Hence, adding the two magnitude categories in addition to pitch-elevation enabled us to investigate the hypothesised advantage for musicians in task varying in cognitive demand, which we hypothesised could reflect on the two measures used (RT and accuracy).*

*Lastly, it is not clear to us how the vertical mental numerical line (particularly with Chinese number words mentally aligned top-to-bottom) is particularly problematic here: the only change in spatial elevation was with the pitch-elevation category and all our visual stimuli were centred horizontally. Moreover, in the manuscript we do shortly discuss the SNARC and SMARC effects. Perhaps there is confusion relating to the word ‘higher’ in our abstract rules, which in this case refers to only a higher value represented, rather than to spatial elevation.*

4. The authors’ prediction is ambiguous: If musical training induces enhanced multisensory integration, should the prediction be larger congruency effect rather than overall better performance for musicians than non-musicians?

**Response to the reviewer:** *We wish to thank the reviewer for raising this interesting question. We have added parts into the discussion section discussing this point (lines 503 – 534).*

*First, we point out that regarding the group difference between congruent and incongruent condition, as far as we know, there are no previous results to indicate whether the advantage grounds on the processing of the congruent or the incongruent stimulus, as the typical studies in the field use d prime as an idex – which cannot attribute the difference on either of the two conditions, but only infer signal detection. We then relate this observation and further discuss it in relation to predictive coding framework* ([Cross‐modal predictive processing depends on context rather than local contingencies - Dercksen - 2021 - Psychophysiology - Wiley Online Library](https://onlinelibrary.wiley.com/doi/full/10.1111/psyp.13811))*. Particularly, we suggest that in the congruent condition, the underlying predictions are not violated, and as the correspondence is suggested to be innate, the predction should (behaviourally) work equally in the two groups (engagning predominantly top-down processing, which is not explicitly trained in musicians). On the other hand, the incongruent condition violates the prediction and hence, engages bottom-up processing routes. This route, in turn, takes advantage of the perceptual learning effects that musicians develop throughout their training, sharpening the perceptual processing of multisensory stimuli. In other words, due to the enhanced multisensory integration, and sharpened perception in relation to such stimuli, they can better identify the violation from the predicted congruency. We also see this effect in relation to the temporal window of integration, where musicians can more easily discriminate out-of-synch stimuli, because of their sharpened temporal window of integration curve* ([Multisensory integration of drumming actions: musical expertise affects perceived audiovisual asynchrony | SpringerLink](https://link.springer.com/article/10.1007/s00221-009-1817-2)).

*We also point out that although our hypothesis was strongly grounded in previous studies, a worthwhile direction for future studies could be to not only look for the overall effects on congruency, but also to look at measures such as the size of the congruency effect.*

5. The experimental design is confusing and hard to follow:
(1) There were four types of stimuli in each visual and auditory stimulus domain. Would it be possible that some trials would be easier (such as using the tones F5 and E6) than other trials (such as using the tones A5 and C6)?

**Response to the reviewer:** *It is indeed true that some of the auditory stimulus pairs had a larger interval between them than others. The same is true to some extent with the visual stimuli (for example with the elevation category in which the physical difference in spatial distance between the two stimuli varied between the trials). The pairs of stimuli were chosen pseudorandomly and both the auditory tones and the visual stimuli were adopted from previous studies by Paraskevopoulos (2012; 2014; 2015) to establish comparability between those studies and the present study – to the extent it is possible despite differences in methodology.*

*Crucially however, the participants heard, saw, and responded to the same stimuli, but in pseudorandom order. Hence, any potential bias caused by varied difficulty across the trials was eliminated.*

*We have added this information explicitly to the manuscript (line 272-274): “This randomization process also enabled the elimination of any potential bias caused by varying intervals between the auditory tones and the visual stimuli.”*

(2) There were two audiovisual stimulus pairs presented sequentially in each trial. Isn’t one pair of audiovisual stimuli sufficient for response?

**Response to the reviewer:** *We do acknowledge that the task the participants were asked to do could have been communicated more clearly. Here, congruency/incongruency was defined by reflecting whether the abstract rule was followed or not. The reviewer is correct in that a pair of audiovisual stimuli is sufficient for estimating congruency, and this is indeed what we tried to communicate in the manuscript.*

*To make the procedure more clear, we have added additional figure (figure 2) showing the detailed experimental procedure in congruent and incongruent conditions for each of the tree audiovisual stimulus categories. With the figure, we have written a detailed legend explaining the figure (and hence the procedure) in what we believe to be clear terms (see also reply above). Moreover, we have edited the methods section to explain the procedure more clearly (lines 250-290).*

*Lines 250-290 now say the following: “Next, the experiment began. The experimental procedure is shown in Fig 2.*

*Enter figure 2 here*

***Fig 2. Examples of the experimental procedure in both congruent and incongruent conditions for each of the tree audiovisual stimulus category.*** *Panels* ***A – B*** *illustrate congruent and incongruent trials in the pitch-elevation category, respectively. Each panel/trial consisted of a single video with the total length of 860 ms. In this video clip, the participant saw two visual stimuli each associated with a sound on a particular pitch. The pitch varied in frequency and the visual stimulus varied in elevation. Congruency was estimated according to an explicitly learned rule, “The higher the spatial elevation, the higher the tone”. Panels* ***C – D*** *illustrate congruent and incongruent trials in the symbolic magnitude-pitch category, respectively. Here, the stimulus varied in value of the number shown and congruency was estimated according to the explicitly learned rule, “the higher the number presented, the higher the tone”. Panels* ***E – F*** *illustrate congruent and incongruent trials in the non-symbolic magnitude-pitch category, respectively. Here, the stimulus varied in the number of circles shown and congruency was estimated according to the explicitly learned rule, “the more dots presented, the higher the tone”. Note that in each category congruency/incongruency could be induced either with the visual or with the auditory part of the stimuli.*

*Each trial consisted of a single video clip (a single panel on Fig 2). The sequence of events in each trial was as follows: the first audiovisual stimulus was presented in the middle of the screen for 400 ms, followed by a short break of 60 ms before the second audiovisual stimulus (400 ms). Each of the audiovisual stimuli consisted of a visual picture (see Fig 1) associated with an auditory stimulus that varied in pitch. The onset time for the auditory stimuli was synchronized with the onset of the visual images. After presenting both audiovisual stimuli (i.e. after presenting a single trial consisting of a pair of audiovisual stimuli), congruency was estimated; the pair of audiovisual stimuli allowed the participants to estimate congruency between the stimuli based on the explicitly learned abstract rules. These rules were “The higher the spatial elevation, the higher the tone”, “the more dots presented, the higher the tone”, and “the higher the number presented, the higher the tone”, depending on the category. The participant were instructed to respond as quickly and accurate as possible with their right hand only by pressing ‘K’ on the keyboard when the corresponding rules were followed, and pressing the button ‘L’ when the rules were not followed. After the response, a 1000 ms blank screen appeared before the next trial. The researcher further instructed the participants verbally, and made sure the instructions were understood properly.*

*There were two experimental blocks, and each block consisted of 180 trials. In each block, there were three audiovisual stimulus categories with 60 trials in each (30 congruent and 30 incongruent). The order of the trials was pseudo-randomized across the participants so that there were not consecutive trials having the same stimulus type. This randomization process also enabled the elimination of any potential bias caused by varying intervals between the auditory tones and the visual stimuli.”*

(3) A figure of experimental procedure would be helpful.

**Response to the reviewer:** *This again is an excellent suggestion. We have added a figure showing the experimental procedure (see fig.2 and the response above).*

(4) How were the hit and false alarm rates defined when calculating d prime?

**Response to the reviewer:** *We have added the following sentence on paragraph 2.4 Analysis (lines 300-304): For the calculations of the d-prime, hits were defined as congruent stimuli correctly identified as congruent, misses as congruent identified as incongruent, false alarms as incongruent stimuli identified as congruent, and correct rejections as incongruent stimuli identified as incongruent.*

6. In Figure 2, there should be 2x3x2 bars, corresponding to the experimental design.

**Response to the reviewer:** *When recording the mistakes, we had several participants who made zero mistakes in some of the audiovisual categories, especially with congruent stimuli, hence causing issues in the d-prime calculations. As our main interest lied in the potential difference between musicians and non-musicians, rather than between the types of visual stimuli, to overcome the issue caused by participants with zero mistakes, we followed the suggestions of Stanislaw & Todorov (1999) and combined the data from several categories before calculating the hit and false-alarm rates (*[*https://link.springer.com/content/pdf/10.3758/BF03207704.pdf*](https://link.springer.com/content/pdf/10.3758/BF03207704.pdf)*). Consequently, when we investigated the accuracy between the visual categories further, we used the raw number of mistakes in the analyses.*

*We have added this information also in the Analysis section which now states (lines 304-309)“As our main interest lied in the potential difference in accuracy between musicians and non-musicians and as several participants made zero mistakes in some of the stimulus categories, instead of calculating the d-prime for each audiovisual category individually, following the suggestions of Stanislaw & Todorov (1999), we combined the data from the three stimulus categories before calculating the hit and false-alarm rates.* *For the further investigation of audiovisual category-wise accuracy, we used the raw number of mistakes.”*

7. Can the better performance (less errors) in musicians than non-musicians simply reflect a better motor control after musical training of instruments?

**Response to the reviewer:** *It is our view that such a suggested advantage in motor control after persistent musical training would first and foremost affect reaction times and not so much accuracy. In other words, if the better performance was due to better motor control, we think we should have seen an advantage in the reaction times first, which we did not observe here. It is especially counterintuitive to think that an advantage would be seen in accuracy measures but not in reaction times, if it indeed were due to better motor control only. Hence, it is unlikely that the better performance was due to better motor control in musicians.*
