# Supplementary material for: The relationship between musical training and the processing of audiovisual correspondences: Evidence from a reaction time task
Source: PLoS One. 2023 Apr 6;18(4):e0282691. doi: 10.1371/journal.pone.0282691 (PMC10079049; doi:10.1371/journal.pone.0282691)
Supplement: Attachment — Submitted filename: Response to Reviewers.docx [file pone.0282691.s002.docx]

**Response to Reviewers**

**Reviewer #1 comments to the authors**

I thank the authors for their very detailed responses and revision. In particular, the van Bastian & Druey paper was interesting, thanks for bringing that to my attention. Please note, though, that it does not appear in the reference list…

All the minor points have been addressed except one (although it was marked as corrected). In the Participants section, the second paragraph, beginning “We applied a standard…”, should be moved to be either the first or second paragraph of the Analysis section, depending on which the authors feel flows better. This is because it is more about prepping the data for analysis than it is about who participated.

I noticed a few errors of English expression in the revision: for example, “our main interest lied” rather than “lay”; and also some typos, for example, “thank” instead of “than”. Please make sure these, and any others, are caught at the proof stage.

Apart from these small items, it looks great!

**Response to reviewer:** *We wish to thank the reviewer for the overall encouraging feedback on both revision rounds, and we do want to apologize for these typos and mishaps that had slipped into the manuscript. We have proof read the manuscript again and corrected the mistakes accordingly. Due to issues in switching to a different reference manager, we had failed to include one of the in-text references in the bibliography. This has now been corrected (added under ‘B’). We have also followed the suggestion of moving the paragraph from the Participants section to a more proper location (under Analysis.*

**Reviewer #3 comments to the authors**

I had the pleasure of reading this interesting manuscript about crossmodal integration in musicians and nonmusicians. As I read this for the first time, I will give a general overview of the manuscript, and then add specific comments. Note that I also read the previous reviews and it seems to me that the authors addressed well all the points raised. My comments will be, however, slightly different from those already mentioned, but will not require any substantial modification of the present manuscript.

First of all I believe that the study itself is well designed, the manuscript reads well and the theoretical background and discussion are sufficiently rich. I have a general advice though: throughout the manuscript, we can often read "the effects of music training": note that very few studies could really prove that the music training causes some improvements in various perceptual/cognitive skills. Most of studies are based on the comparison of adult musicians and nonmusicians. This is not sufficient to talk about cause/effect relationships. I encourage the authors to talk about "association with the music training" instead of "effects".

Then, in the discussion, clearly there is space of mentioning why the authors believe it is reasonable to consider the music training as the cause. But it is still an intepretation, and the authors should acknowledge, perhaps in the limitation section, that the present study cannot infere any cause-effect relationship.

This is particularly true (and here I suggest to add another limitation) as there was no general control of cognitive abilities of the two groups. For example, the speed of processing subtests of the WAIS-IV could have been informative in explaining why there were no differences between groups in the RTs. Having no control tasks, cannot exclude that the two groups were different in terms of general cognitive abilities. Also, I did not read any information about years of education. Was this variable collected? Are the group different in years of education?

**Response to reviewer:** *The authors would like to sincerely thank the reviewer for the positive and very constructive and insightful feedback. We absolutely agree with the point regarding causality: it is indeed the case that we cannot demonstrate causality in a decision reaction-time task such as in the present manuscript. We do not wish to claim that such causality is demonstrated, although we do recognise that we have used unclear language and semantics that easily reads as we indeed wish to suggest such causal relationship. We have changed the language throughout the manuscript to refer to relationship/association/link between musical training and advantages in multisensory integration, as suggested. We also added a paragraph in the discussion section stating this explicitly. In that paragraph we also suggest that future studies should use a control task/tasks and collect more extensive background knowledge from the participants to have stronger grounding for suggesting causality between musicianship and observed advantages (lines 568-576).*

Again on the groups differences. I see that the inclusion criteria for nonmusicians was not having formal training outside from the mandatory classes at school. Did the authors checked whether the nonmusicians could have, anyway, learnt to play an instrument as self-taught? For instance, as they "self-identified as having no musical expertise", was musical expertise defined as having received formal lessons? This is important, because one could still play a bit as an amateur but consider him/herself not an expert. What were the exact questions asked to gather information about musical expertise?

**Response to reviewer:** *This is again an important point. We did ask for any musical experience from the participants, and as we mention in Participants section under Materials and Methods, we disregarded one participant due to having been playing the piano between the ages of 7 to 12 years old. Self-identified musical experience here was based on asking if the participants played any instruments, composed music on a computer, or had taken any lessons for any instruments at any point in their life. Any continuous lessons or practicing excluding the compulsory music lessons in elementary/high-school resulted in exclusion from the experiment. To be more precise, we have added this last sentence into the manuscript (lines 181-182).*

Similarly, I find the range of musical expertise in the musician group quite large. Studies including musicians usually have stricter criteria (e.g., 7-8 years of training at least). 3 years seems not a enough. Did the authors check at least whether the musicians were active at the moment of the testing? Because if an individual had 3 years of experience but stopped to play 10 years before, well, this wouldn't really qualify as being a musician in my opinion. I encourage the authors to add more details about the two groups, and if these details are not available, to include the criteria used to create the two groups as a possible limitation (that might also explain different results from previous studies, perhaps).

**Response to reviewer:** *We indeed did ask whether the musicians were currently active and that was one of the inclusion criteria and we have added this detail into the manuscript as well. We also asked the participants their mean training time per week, which varied between 1 to 6 hours, but as we didn’t have this information for all of the musicians, we did not include it in the manuscript. Moreover, only 2 musicians reported having less than 6 years of training, hence we do not believe the more flexible definition of musicianship played a significant role in the results (in comparison to other studies in the field). We have added more details to the manuscript regarding the definition of musicianship.*

*We also noticed a mistake in the mean number of musical education and updated the value to the correct mean after participant exclusion.*

I know that the athors provide already many analyses, but perhpas it could be interesting to look at the correlations between years of musical expertise and RTs/accuracy, as the range is very wide?

**Response to reviewer:** *As the manuscript already includes a number of analyses, we are hesitant to add anymore into the mix. We also feel that our analyses already cover these questions to an extent: it was precisely our hypothesis that reaction times and number of mistakes made would decrease as a function of musical expertise which could be captured with number of years of training.*

*However, based on this feedback we did run the correlation analyses suggested. All correlations with reaction times were non-significant, while all correlations with the number of mistakes made in each category were statistically significant with all correlations being negative (i.e. less mistakes made when musical education increased). This is not surprising given our previous results indicating that musicians made fewer mistakes than non-musicians (with training of 0 years), and that no differences were found in reaction times.*

*These results remained the same regardless of whether we correlated only musicians or included non-musicians (with training years of zero) into the sample. Hence, respectfully, we do not think including them would add a lot of value into the manuscript.*

Finally, I do not see in the manuscript any statement or link for data availability. My apologies if this will appear later on, in any case, I think it would be great to provide a link where readers can access the dataset.

**Response to reviewer:** *During the submission process we mentioned that the data will be made available upon publication. It is now online and can be found at https://gin.g-node.org/rihalai/Crossmodal_Correspondences*


Some specific details I noticed:
-Figures: Figure 1 and 2 seem very low resolution.

**Response to reviewer:** *We agree figure 1 had low resolution and we have re-made it with higher resolution. The resolution in figure 2 is better and is dictated by the resolution of the original stimuli images.*

-page 4, line 85: "audiovisual congruency effects" I suggest adding a definition of what these effects are in practice (e.g.,higher accuracy in identifying congruent pairs, shorter RTs, etc.?), as there might be different things to which the authors are referring.

**Response to reviewer:** *These studies are discussed in detail in the very next paragraph (starting from line 87). This was not very clear, though, and we have now edited the manuscript such that the new paragraph starts from the line first mentioning the “audiovisual congruency effects” and is followed by the more detailed discussion of those experiments.*

-page 4, line 94: "at discriminating the stimuli" This reads a bit vague, I suggest clarifying what the task required.

**Response to reviewer:** *Agreed. We changed this to refer to the task of judging whether the stimuli were congruent with the rule.*

-page 4, line 97,98: " pitch-elevation stimuli" and "non-symbolic magnitude" are not clear yet in the introduction, I suggest to define what are these types of stimuli, otherwise the reader will understand it only in the method section

**Response to reviewer:** *We have edited the text to refer to spatial elevation, which is mentioned before, and generally to visual representations of magnitudes (in conjunction with pitch).*

-page 5, line 114: here the "detection reaction time task" is also a bit vague, had the participants to respond to congruency again?

**Response to reviewer:** *We have added more details of the task into the description. It now says “…with a detection reaction time task, in which the participants were instructed to click a mouse button immediately upon perception of auditory, tactile, or simultaneous audio-tactile stimuli, and reported that*…” *(line 114-118).*

-page 5, line 117: Apologise if it is my mistake, but by reading the description of the study by Bidelman et al., it seems that the musicians had less frequently the audiovisual illusion. Does this mean that they integrated better the stimuli? Because intuitively, I would say that if they integrated them better, they would suffer the illusion more, not less. Having less frequently the illusion (or with shorter - less detectable - durations) might indicate, to me, that they could segregate better the two types of stimuli, not integrate them. But I might misunderstand what it's written.

**Response to reviewer:** *This is a fair question. We discuss Bidelman et al. (2016) paper as an example of observed processing speed advantage in musicians over non-musicians, and this was indeed one of the results of Bidelman et al. (i.e., musicians were faster at making their response than nonmusicians and that musicians were not only more accurate at processing concurrent audiovisual cues but considerably faster at judging the composition of audiovisual stimuli).*

*In addition, they did observe musicians to have lower susceptibility for perceiving the illusory effect. In the paper, Bidelman et al. talk about “considerably more refined binding of auditory and visual cues” and “..musicians have enhanced multisensory integration and are better able to accurately parse audiovisual cues”. They conclude that musical experience “improves multimodal processing and integration of multiple sensory systems”. On the other hand, in their paper, they cite other results showing age-related increased multisensory integration “.. as evidenced by broader temporal binding window”. We take it that it is their position, that taken together, these results indicate a more refined, improved multisensory integration/processing (whereas looking at the narrower temporal binding window in musicians alone would probably be characterized differently if discussed in isolation).*

*In the manuscript, we were careful not to talk about “increased multisensory integration” in relation to these results, but rather, “improved multisensory integration”. We have further edited the part to refer to a more refined, improved integration of multiple sensory systems in a domain-general manner.*

-Page 7, line 150: I'm not a native speaker but starting a paragraph with "therefore" reads strange.

**Response to reviewer:** *We have changed this to “Hence”.*

- Page 7, line 162: I suggest writing that the power analysis is explained later on, otherwise at a first glance one could wonder why no details about it are reported.

**Response to reviewer:** *Corrected.*

-Page 8, line 170: "made a mistake". How is the mistake defined here? Because later on, mistakes (wrong answers) are taken into account in the analyses, so I believe that this is a different type of mistake.

**Response to reviewer:** *These mistakes were indeed wrong answers (analysed later on). Here, we refer only to the reaction time measure: for the calculations of the reaction times, we only used the recorded data from the correct responses. Note that this paragraph is now moved under the Analysis-section, as per the suggestion of Reviewer #1.*

-Page 17. I find it a bit strange to read that there is a difference between musicians and nonmusicians in numbers of mistakes in the incongruent trials, but then, in the last paragraph with this analysis there is no difference between groups in mistakes in congruent and incogruent conditions. (see line 382, "irrespective of musicianship"). Are these two results a bit in contraddiction? If the musicians have less mistakes in the incogruent conditions, I would expect an interaction, not an overall effect of congruency.

**Response to reviewer:** *Here, the last result did not look at group-differences at all (between musicians and non-musicians). Rather, we analysed the overall number of mistakes made (musicians and non-musicians combined) in all congruent trials and in all incongruent trials, and found a large difference indicating more mistakes in incongruent trials (as expected). This difference was there irrespective of musicianship (i.e. for both groups combined). We can understand that by mentioning musicianship here may be confusing as we are looking at all participants together, and hence, we have removed that part from the paragraph (“irrespective of musicianship”).*

-page 19, line 410, 411: Can the authors report the statistics (at least the p-values, as before) for the post-hoc significant comparisons?

**Response to reviewer:** *We have added the p-values for this estimated marginal means interaction. We have also corrected the language in this paragraph, and related the interaction more directly to elevation category.*

-page 20, line 416: I think that here mentioning "effects of long-term training" is quite tricky for the reasons mentioned before: (1) there is no way to understand any effect of musical training with the present study, (2) speaking about long training with a inclusion criteria of >3 years seems a bit optimistic).

**Response to reviewer:** *This is now corrected to “.. explore the association between long-term musical training and bimodal sensory integration”.*

I think that if these minor details are clarified, the manuscript will be then ready to be publicated.

**Response to reviewer:** *We appreciate all the feedback and feel that the manuscript is much stronger after editing it accordingly.*
